# Relapsing polychondritis-associated meningoencephalitis initially presenting as seizure: a case report and literature review

**DOI:** 10.3389/fneur.2023.1265345

**Published:** 2023-11-14

**Authors:** Dan Zhang, Jiamin Shi, Xinhua Zhang, Jin Wang, Yuquan Shao

**Affiliations:** ^1^Department of Neurology, Sir Run Run Shaw Hospital, Zhejiang University School of Medicine, Hangzhou, China; ^2^Department of Neurology, Jinhua Wenrong Hospital, Jinhua, China; ^3^Department of Rheumatology, Sir Run Run Shaw Hospital, Zhejiang University School of Medicine, Hangzhou, China

**Keywords:** relapsing polychondritis, inflammatory meningoencephalitis, seizure, neuroimmune disease, immunosuppressants

## Abstract

**Background and purpose:**

Relapsing polychondritis (RP) is a rare rheumatologic disorder that may affect the neurological system with various presentations. In this study, we present a case and summarize the clinical characteristics of RP-associated meningoencephalitis.

**Case presentation:**

A 48-year-old man presented with first-ever seizures that were well controlled by valproate. Physical examination results were unremarkable, except for binaural deformation. The initial brain magnetic resonance imaging (MRI) without contrast and electroencephalogram (EEG) findings were normal. However, the patient subsequently developed recurrent fever, scleritis, headache, lethargy, and left arm paresis. Repeated brain MRI with contrast demonstrated increased enhancement of the pia mater and abnormal diffusion-weighted imaging (DWI) signals in the bilateral auricles. The cerebrospinal fluid (CSF) analysis showed 2 leukocytes/μL, 736.5 mg/L of protein, and no evidence of infectious disease or autoimmune encephalitis. Meningoencephalitis secondary to RP was considered. The patient's condition improved significantly and quickly with the administration of dexamethasone (10 mg per day). Oral methylprednisolone was continued, and the patient remained well without relapse during the 9-month follow-up period.

**Conclusion:**

RP-associated meningoencephalitis is rare but fatal. Although symptoms vary, red or deformed ears remain the most common and suggestive features. Non-specific parenchymal changes and/or meningeal enhancement can be observed on brain MRI scans. CSF lymphocytic pleocytosis with mild protein elevation was observed in most patients.

## Introduction

Relapsing polychondritis (RP) is a rare rheumatological disorder characterized by recurrent inflammation and destruction of cartilage throughout the body (1). Patients can present with multiple sets of complaints, including ear pain, nasal pain, hoarseness, throat pain, arthritis, episcleritis, scleritis, and less frequently, cardiac, neurological, and renal diseases (2). Neurological involvement is rare but fatal in RP (3) and is difficult to diagnose due to its rare frequency and variable presentation. Encephalitis, meningitis, meningoencephalitis, myelitis, polyneuritis, seizures, stroke, cerebral aneurysm, and headache have also been reported (2, 3). In this study, we report a case, review the literature, and summarize the clinical characteristics of meningoencephalitis associated with RP.

## Methods

**Figure 1:**
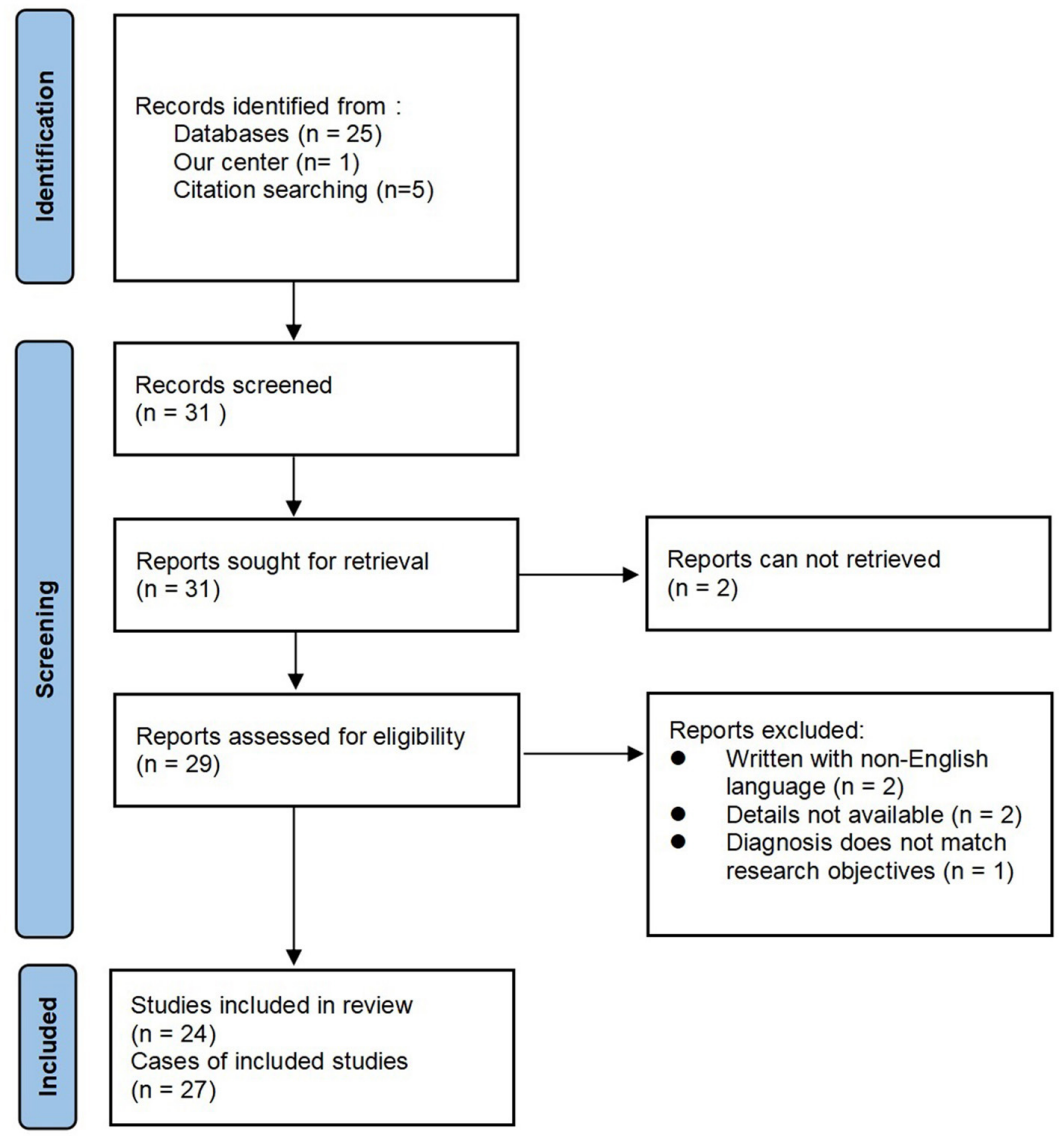
Adapted PRISMA 2020 flow diagram.

We report a case of meningoencephalitis associated with RP from the Sir Run Run Shaw Hospital. In addition, we performed a literature search of the online database PubMed in July 2023 using Endnote with the terms “relapsing polychondritis” combined with “meningoencephalitis,” without time frame restrictions. The reference lists of the selected articles were screened for additional relevant articles. Owing to confusion and overlap in the diagnoses of encephalitis, meningitis, and meningoencephalitis, only articles that explicitly stated the diagnosis of meningoencephalitis were included in this study. Articles without full text, those written in languages other than English, and those without sufficient clinical data were excluded. The adapted Preferred Reporting Items for Systematic Reviews and Meta-Analyses (PRISMA) flow diagram is shown in Figure 1.

The full texts of potentially relevant literature were reviewed by two authors (DZ and JMS) independently. Disagreements were resolved through discussion and consultation with a third author (YQS or JW). Clinical information, including age, sex, neurological symptoms, neuroimaging findings, cerebrospinal fluid (CSF) analysis, electroencephalogram (EEG) findings, treatment, and prognosis, was extracted from each article (Table 1).

**Table 1:** Reports of patients with relapsing polychondritis and meningoencephalitis.

**References**	**Age-Sex**	**RP history**	**Ear sign**	**Fever**	**Neurological symptoms**	**Seizure (type)**	**Meningeal**	Parenchyma^*^	**CSF Pleocytosis**	**CSF protein (mg/L)**	**EEG**	**Immunotherapy^#^**	**Prognosis**
Ohta et al. (4)	57 M	N	+	+	Vertigo, hearing loss, headache, memory loss, anxiety, depression	-	-	T	119 (L85%)	860	Diffuse slow wave	mPSL-P, PSL	Improved
Fujiki et al. (5)	45 M	N	+	+	Headache, confusion, euphoria, disorientation forgetfulness	-	-	T	800 (L94%)	860	NA	**PSL**	Improved
	62 M	N	+	-	Memory loss, confusion, euphoria, hearing loss, amnesic	-	-	T	2,400 (L83%)	46,000	NA	mPSL-P, **PSL**	Not improved
Ota et al. (6)	57 M	1 year	+	+	Vertigo, hearing loss, weakness, seizure, delirium, personality change	GTCS	+	-	1,056 (L15%)	690	NA	PSL, mPSL-P, **PSL**	Not improved
Kao et al. (2)	40 M	N	+	+	Headache, confusion	-	-	T, basal	1,500 (L17%)	850	Intermittent slow in the left	mPSL-P, PSL	Improved
Erten-Lyons et al. (7)	51 M	1 year	+	-	Progressive dementia, anxiety, depression, insomnia	Myoclonus	-	Peri-V, DWM	39 (L65%)	890	Normal	PSL, CTX	Declined, died
Imamura et al. (8)	76 F	N	+	+	Decreased speech and voluntary behavior, impaired consciousness, hearing loss	-	-	WM	+	↑	NA	mPSL-P, MTX, CTX	Declined, died
Wang et al. (9)	54 M	N	+	+	Bipolar disorder, headache, memory loss, hallucination, hearing loss	-	-	WM	800 (L95%)	600	Background slow wave	DXM, CTX + mPSL, mPSL-P, **PSL** **+** **Aza**	Improved
	44 M	N	+	-	Memory loss, anxiety	-	-	T	190 (L90%)	570	NA	mPSL-P, **PSL** **+** **Aza**	Improved
	52 M	N	+	+	Headache, memory loss, difficulty in communicating, deafness, gait disorders, urinary incontinence	-	-	-	230 (L29%)	510	NA	DXM, mPSL-P + IVIG, **PSL**, mPSL-P	Improved-recur-improved
Choi and Lee (10)	68 F	2 months	+	-	Dysarthria, impaired language function	-	+	WM	100 (L67%)	416	Moderate (slow wave)	PSL + MTX, mPSL-P, PSL + MTX, **PSL**	Improved
Garcia-Egido et al. (11)	57 M	N	+	+	Headache, seizure, confusion	GTCS	-	Peri-V	700 (L98%)	750	NA	PSL, PSL + CTX/MTX, **PSL** **+** **Infliximab**	Recur-improved
Fujiwara et al. (12)	60 M	N	+	NA	Headache, speech impediment, acalculia, agraphia, right-left disorientation, mild right hemiparesis	Focal	+	-	Normal	Normal	NA	**PSL**	Improved
Prinz et al. (13)	63 ?	NA	+	NA	Headache, neuropsychological retardation	-	-	+	Lymphocytic	NA	NA	NA	NA
Nara et al. (14)	39 M	N	+	-	Headache, psychiatric symptoms	-	-	DWM	19 (L100%)	710	NA	PSL, mPSL-P, **PSL**	Improved
Baba et al. (15)	72 M	2 years	+	+	Somnolent, dysarthria, cognitive, hemi spatial neglect	-	-	WM	781 (L13%)	5,820	NA	DXM, PSL, **CTX**	Improved
Jeon (16)	48 F	2 months	+	-	Headache	-	-	C, basal	71 (L58%)	866	NA	mPSL-P	Declined, died
Tsai et al. (17)	44 M	N	+	-	Headache, seizure, confusion, hallucination	+	-	WM	Lymphocytic	NA	NA	mPSL-P + CTX, PSL + CTX, **Aza**	Improved
Ducci et al. (18)	69 M	N	+	-	Ataxia, paraparesis, tinnitus, vertigo, confusion, tremor	-	+	-	71 (L97%)	697	NA	PSL, **MTX**	Improved
AI-Tabbaa and Habal (19)	25 M	N	+	-	Hallucination, anxiety, depression	-	+	Peri-V, WM	21 (L81%)	NA	NA	mPSL-P, CTX, PSL, rituximab, **PSL** **+** **MTX**	Declined
Cao and Zhang (20)	64 M	N	+	+	Headache, dull response, unsteady gait, paralysis, cognitive decline	-	+	Peri-V	520 (L40%)	770	NA	PSL, mPSL-P + MTX, **MTX**	Improved
Anada et al. (21)	65 M	N	-	+	Drowsy, urinary retention	-	-	Basal	NA	NA	NA	mPSL-P, **PSL**	Improved
Lin et al. (22)	38 M	2 years	+	-	Abnormal behavior, slurred speech, memory loss/aphasia, weakness, impaired vision, diplopia, insaneness	-	-	T, basal	15	533	NA	PSL, mPSL-P, mPSL-P + IVIG, **PSL** **+** **MMF**	Improved
Matsumoto et al. (23)	61 M	N	+	+	Headache, confusion, delirium, unable to communicate	Myoclonus	+	basal	189 (L52.6%)	1,990	Poorly organized	DXM, mPSL, **PSL** **+** **MTX**	Improved
Lokken and Wang (24)	36 M	N	+	-	Headache, vision loss	-	+	T	NA	NA	NA	Adalimumab + PSL	Died
Yokota et al. (25)	49 M	4 years	+	-	Hearing loss, cognitive impairment	-	-	T, DWM	Normal	540	Diffuse slow wave	PSL, mPSL-P, PSL + CTX, **PSL** **+** **MTX**	Improved
Our case	46 M	N	+	+	Seizure, headache, somnolent, slow response, left arm weakness	Focal	+	-	Normal	737	Diffuse slow wave	DXM, **mPSL**	Improved

The cases above are listed according to the published date. Aza, azathioprine; CSF, cerebrospinal fluid; CTX, cyclophosphamide; DXM, dexamethasone; EEG, electroencephalogram; GTCS, generalized tonic-clonic seizure; IVIG, intravenous immunoglobulin; L, lymphocytic; mPLS, methylprednisolone; mPLS-P, methylprednisolone pulse; MMF, Mycophenolate mofetil; MTX, methotrexate; N, no; NA, not available; PLS, prednisolone; RP, relapsing polychondritis. ^*^Basal, basal ganglia area; C, cerebellum; DWM, deep white matter; Peri-V, periventricle region; T, temporal lobe; WM, white matter. ^#^The bold font shows the treatment that the patient was still receiving at the time of the literature report. ^+^Means positive for this item, while ^−^ means negative. ^?^Means sex was stated for this patient. ^↑^Means protein elevated in this patient, but exact numerical value was not available.

## Results

### Case presentation

A 48-year-old man presented to the emergency department with cluster seizures. Following intravenous injections of diazepam and valproate, the patient gradually became fully oriented, alert, and afebrile. Cranial computed tomography (CT) and CT angiography performed in the emergency department were normal. The patient was admitted to the neurology department to evaluate the etiology of the first-ever seizures.

**Figure 2:**
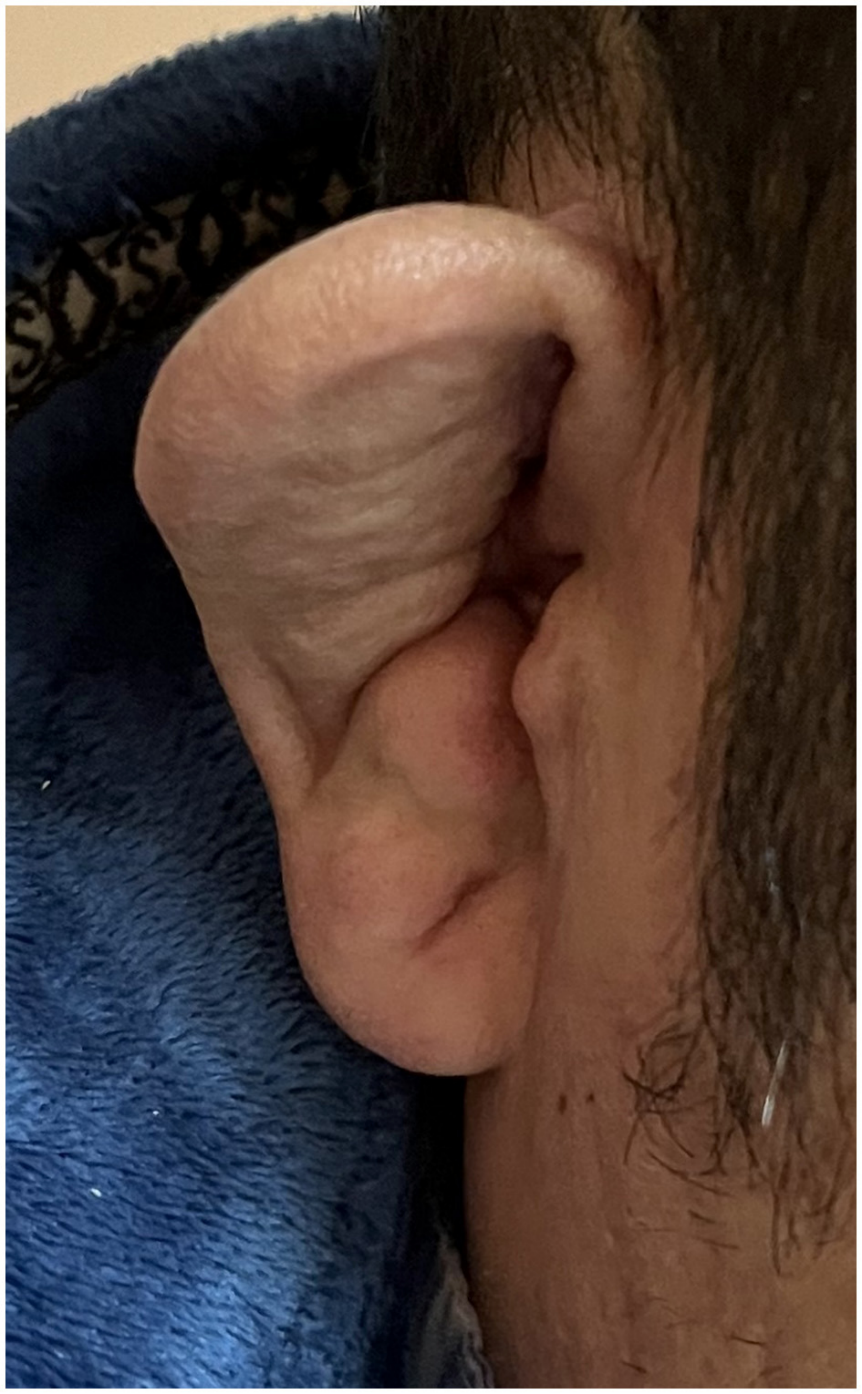
Deformed ear.

Neurological examination was unremarkable on admission; however, binaural deformation was present (Figure 2). In the past year, the patient experienced recurrent auricular pustules, which improved after treatment at another hospital, but auricular hypertrophy and deformation remained. Laboratory examination results were as follows: 6,900/μl of white blood cell count (neutrophils 75.2%), 26.8 mg/L of C-reactive protein (reference value < 6.0 mg/L), 315.8 IU/mL of thyroglobulin antibody (TGAb) (reference value 0.0–4.1 IU/mL), and 245.20 IU/mL of thyroid peroxidase antibody (TPOAb) (reference value 0.0–5.6 IU/mL). Thyroid-stimulating hormone (TSH), T3, T4, tumor markers, and hepatic and renal functions were normal. The antinuclear antibody series, antiphospholipid antibodies, vasculitis series, serum human immunodeficiency virus (HIV) serology, and rapid plasma reagin (RPR) tests were negative. Brain magnetic resonance imaging (MRI) without contrast and EEG were unremarkable. On the fourth day of hospitalization, the patient developed a transient low fever with spontaneous remission the next day. Lumbar puncture was suggested but refused by the patient, who was then discharged with oral valproate therapy.

After 18 days of initial discharge, the patient was readmitted because of fever, headache, and left arm paresis. During the past half a month, the patient had recurrent mild fever, with a body temperature fluctuating between 37 and 38°C, accompanied by headache. The patient reported no cough, expectoration, frequent urination, urgency, abdominal pain, diarrhea, or seizure recurrence. The white blood cell count was 12,100/μL (neutrophils 83.1%), and the C-reactive protein was 18.3 mg/L, as tested in another hospital. Ceftriaxone was intravenously administered for more than 4 days without any relief. On the day before admission, left arm numbness and weakness occurred, which lasted for half an hour, followed by slurred speech and difficulty in expression. Considering that stroke could not be ruled out, aspirin and clopidogrel were administered along with ceftriaxone, acyclovir, and valproate.

**Figure 3:**
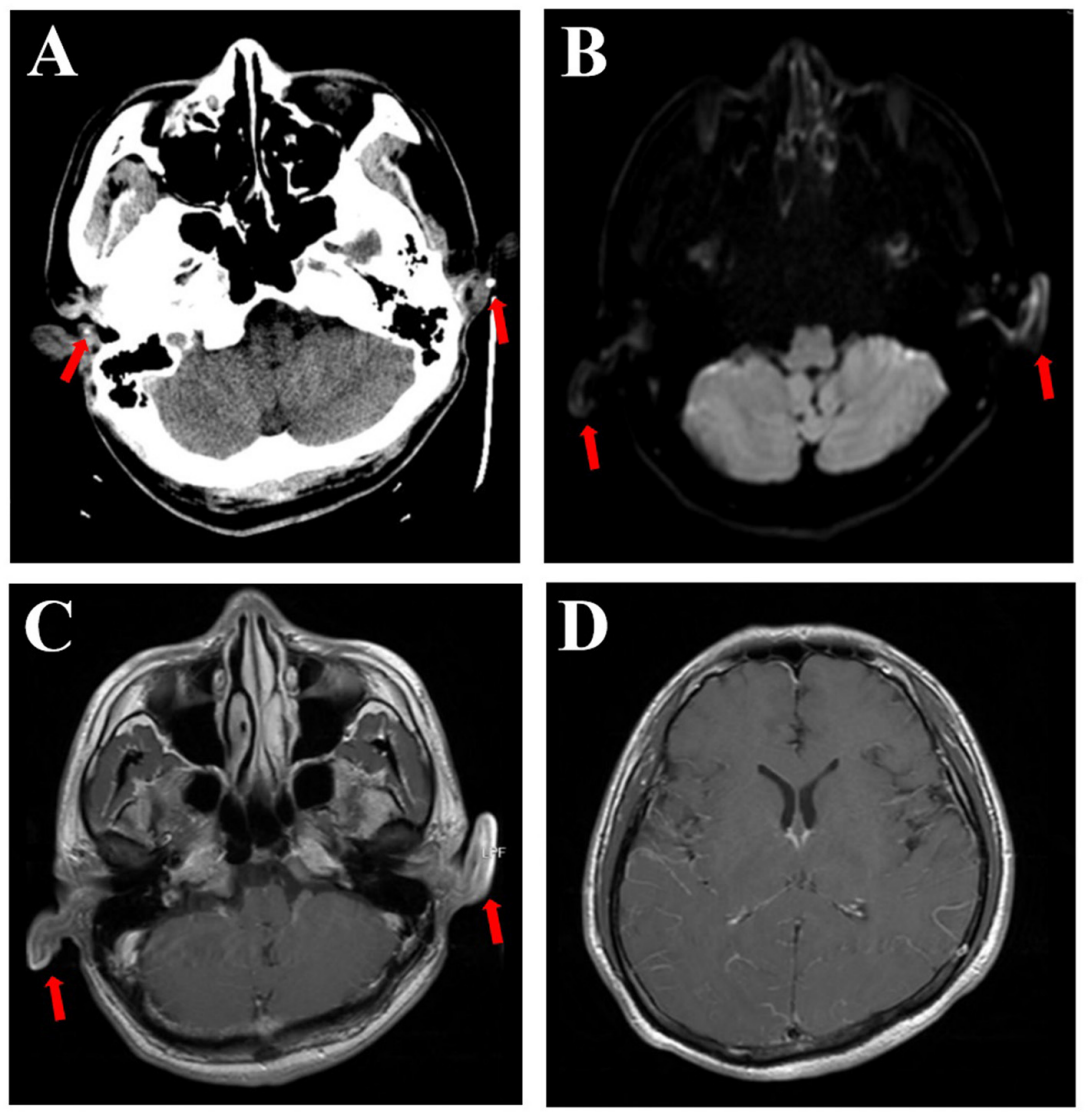
Brain CT scan showing calcification (arrows), which can be seen in the polychondritic ears **(A)**. DWI shows abnormal signals in the bilateral auricles (**B**, arrows). T1-weighted MRI with contrast showed abnormal signals and thickening of both ears (**C**, arrows) and increased enhancement of the pia mater **(D)**.

Upon admission, vital signs were as follows: body temperature 38.9°C, pulse 83 bpm, respiratory rate 19/min, and blood pressure 123/72 mmHg. The patient was somnolent but arousable and responded slowly. The cranial nerve examination was normal, except for red eyes. Motor system examinations revealed left arm paresis graded 4+ on the Medical Research Council (MRC) scale. Kernig's signs were positive, with no neck resistance. There were no abnormalities in the reflex, sensory, or coordination systems. Laboratory tests showed a white blood cell count of 14,300/μL (neutrophils 88.6%), C-reactive protein of 17.6 mg/L, erythrocyte sedimentation rate of 60 mm/h, and procalcitonin within the normal range. Aspirin and clopidogrel were discontinued upon admission to the hospital ward. Ceftriaxone and acyclovir were continued. Lumbar puncture was performed the day after admission, with an opening pressure of 175 mmH_2_O. CSF analysis showed 2 leukocytes/μL, 736.5 mg/L of protein, and 50.0 mg/dL of glucose (103 mg/dL of simultaneous blood glucose). CSF adenosine deaminase, occult blood, Gram stain, acid-fast staining, ink staining, tuberculosis, and herpes virus antibodies were all negative. CSF cytology revealed a mixed-cell inflammatory response. CSF metagenomic next-generation sequencing revealed the presence of streptococcus. Contrast-enhanced brain MRI demonstrated an increased enhancement of the pia mater (Figure 3), with no abnormal signals in the brain parenchyma. Abnormal DWI signals were observed in the bilateral auricles (Figure 3). EEG revealed a moderate slow-wave change, which was more pronounced on the right side. Both peripheral blood and CSF autoimmune encephalitis antibody spectrum tests, including anti-N-methyl-D-aspartate receptor (anti-NMDAR), anti-leucine-rich glioma-inactivated 1 (anti-LGI1), anti-contactin-associated protein-like 2 (CASPR2), anti-gamma aminobutyric acid-B receptor (GABA_B_R), anti-α-amino-3-hydroxy-5-methyl-4-isoxazolepropionic acid receptor 1 (anti-AMPAR1), anti-AMPR2, anti-immunoglobulin-like cell adhesion molecule 5 (anti-IgLON5), anti-dipeptidyl-peptidase-like protein 6 (anti-DPPX), anti-glutamic acid decarboxylase 65 (anti-GAD65), anti-metabotropic glutamate receptor 5 (anti-mGluR5), anti-glycine receptor (GlyR), and anti-R2R, were all negative. The T-SPOT.TB test and IgG4 were also negative.

Although ceftriaxone was administered before admission, consultation with the Infectious Disease Department suggested that bacterial meningitis was not considered. Therefore, meningoencephalitis secondary to RP was suspected. Consultation with the Rheumatology Department yielded a similar opinion as ours.

The patient gradually became drowsy during hospitalization. The paresis of the left arm became severe. His son, who was taking care of him, also developed fever. The coronavirus disease 2019 (COVID-19) nucleic acid test results were positive. Considering the COVID-19 infection, dexamethasone (10 mg/day) was administered intravenously, along with symptomatic treatment for COVID-19. After 2 days of steroid therapy, there was a significant improvement in drowsiness and paresis, with no seizure recurrence. Dexamethasone (10 mg/day) was given for 2 weeks, followed by methylprednisolone at 56 mg/day, which was gradually tapered during follow-up. A follow-up EEG performed 6 months after disease onset was normal, and valproate was therefore discontinued. During the 9-month follow-up, the patient remained well without neurological disorders while continuing with methylprednisolone (current dose, 12 mg/day).

### Literature search result

The literature review yielded an additional 26 cases of meningoencephalitis associated with RP from 24 articles (Table 1). There were 22 men and 4 women, and 1 patient did not state the sex. The mean patient age was 53 (range, 25–76) years (Table 2). Only 26% (7/27) of the patients had been diagnosed with RP before the reported neurological event. Most patients (95%, 26/27) had red or deformed ears, while fever was reported in 52% (13/25) of the cases. Various symptoms were reported, including headaches (56%, 15/27), psychiatric symptoms (44%, 12/27), memory or cognitive disturbance (41%, 11/27), cranial nerve symptoms (37%, 10/27), confusion (33%, 9/27), and weakness (19%, 5/27). Brain MRI revealed the presence of parenchymal lesions in 81% (22/27) of the patients, while only nine cases indicated meningeal enhancement. In CSF analysis, pleocytosis was observed in 88% (22/25) of the patients, with lymphocyte predominance in 75% (15/20). CSF protein elevation was observed in 95% (21/22) of the patients, of whom 85% (17/20) had protein levels between 450 and 1,000 mg/L. Slow-wave changes were the most common EEG result (75%, 6/8), although very few cases have been reported. Steroids were the mainstay of treatment, with 52% (14/27) of the patients receiving immunosuppressive therapy combined with steroids. Improvement was achieved in 73% (19/26) of the patients, with a mortality rate of ~15% (4/26).

**Table 2:** Clinical characteristics of patients with meningoencephalitis complicated in relapsing polychondritis.

	***n/N* (%)**
Mean age, y (SD)	53 (12)
**Sex**	
Male	22/26 (85%)
Female	4/26 (15%)
Previously diagnosed with RP	7/27 (26%)
**Symptoms and signs**	
Cauliflower ear	26/27 (96%)
Fever	13/25 (52%)
Seizure	7/27 (26%)
Headache	15/27 (56%)
Psychiatric symptoms	12/27 (44%)
Memory/cognition disturbance	11/27 (41%)
Cranial nerve symptoms	10/27 (37%)
Confusion	9/27 (33%)
Weakness	5/27 (19%)
**Brian MRI**	
Meningeal enhancement	9^*^
Parenchymal lesion	22/27 (81%)
CSF pleocytosis	22/25 (88%)
>500/μL	8/25 (32%)
Lymphocyte predominant	15/20 (75%)
CSF protein elevation	21/22 (95%)
450~1,000 mg/L	17/20 (85%)
**EEG**	
Slow wave change	6/8 (75%)
**Prognosis**	
Improved	19/26 (73%)
Not improved or declined	7/26 (27%)
Mortality	4/26 (15%)

^*^Percentage was not calculated as many case reports did not state whether brain MRI was performed with or without contrast. For patients with multiple CSF study results, the one performed during the reported neurological event, or the latest one before steroid use, was included in the analysis. Psychiatric symptoms included anxiety, depression, bipolar disorder, hallucination, euphoria, and delirium. CSF, cerebrospinal fluid; EEG, electroencephalogram; MRI, magnetic resonance imaging; RP, relapsing polychondritis; SD, standard deviation.

## Discussion

Currently, the diagnosis of RP is established clinically by excluding differential diagnoses. There are three diagnostic criteria for RP. Rose et al. evaluated the sensitivities of these different criteria and modified the Michet criteria in 2018 (Table 3). Based on recurrent auricularis, scleritis, and response to steroids, our case fulfilled the Damiani and Levine criteria and the Modified Michet criteria for RP.

**Table 3:** Diagnostic criteria of relapsing polychondritis.

McAdam et al. (26) (three out of six)	1) Recurrent bilateral auricular chondritis 2) Non-erosive seronegative inflammatory polyarthritis 3) Nasal chondritis 4) Ocular inflammation 5) Respiratory tract chondritis 6) Cochlear and/or vestibular damage
Damiani and Levine (27) (any of these)	1) Three out of six McAdam's criteria 2) One out of six McAdam's criteria + positive histology 3) Two out of six McAdam's criteria + response to corticosteroid or dapsone
Michet et al. (28) (any of these)	1) Inflammation in two out of three cartilages: auricular, nasal, and laryngotracheal 2) Inflammation in one of the above + meeting two other signs of ocular inflammation, hearing loss, vestibular dysfunction, or seronegative inflammatory arthritis
Modified Michet's criteria (any of these) (29)	1) Inflammation in two out of four sites: auricular, nasal, laryngotracheal, and ocular inflammation 2) Inflammation in one of the above + meeting two other signs hearing loss, vestibular dysfunction, seronegative inflammatory arthritis, or dermatologic and cardiovascular manifestation

Neurological involvement in RP is rare and affects ~3% of patients; however, it is an important cause of death (3). The pathogenesis of central nervous system (CNS) involvement in RP is still unknown but appears to be related to autoimmunity. Cerebral and meningeal vasculitis have been reported in autopsy cases of RP with meningitis or meningoencephalitis (2, 4, 9). The diagnosis of CNS complications in RP is mainly clinical and challenging because of the varied clinical features.

The onset of meningoencephalitis can precede the diagnosis of RP or occur during a relapse of RP. In our study, we found that only 26% of patients had been previously diagnosed with RP. The diagnosis of RP was first confirmed because of the reported neurological events in most patients. In this situation, the diagnosis of RP is even more challenging owing to its complicated manifestations and the need for additional differential diagnoses. Our study summarizes the clinical characteristics of meningoencephalitis in RP to facilitate its recognition, prompt diagnosis, and treatment.

Previous studies have revealed that the peak age of onset of RP is in the fifth decade of life (2, 9). The mean age of patients with meningoencephalitis in our study was 53 years, which is in line with the age characteristics of RP onset. RP affects both men and women with an equal distribution (2). However, in our study, we found that RP-associated meningoencephalitis predominantly affected men, which is consistent with a previous study (25). It is reported that RP-associated myelodysplastic syndromes are mostly seen in men >60 years of age (1). Another study found that male patients had a higher prevalence of hearing loss, vestibular disorders, and uveitis events (30). However, since the etiology and pathophysiology of RP remain unclear, there is currently insufficient evidence to explain the reasons for this sex distribution difference.

A red or deformed ear is one of the most common and suggestive features of RP and is present in up to 90% of patients during the course of the disease (1). In our study, this sign was present in 96% of the included patients. For the patient in our center, recognition of the deformed ear ultimately led to the diagnosis. The mean delay in RP diagnosis is 2.9 years (9). Our patient had recurrent auricular pustules for more than 1 year before this event without a clear diagnosis. However, the patient may not have met the diagnostic criteria because of the limited involvement of anatomical locations at that point. However, it is crucial to recognize this special and suggestive sign and delve deeply into the diagnosis.

Neurological symptoms are diverse in this group of patients, with headaches, psychiatric symptoms, memory disturbance, hearing loss, and confusion being relatively common. Seizures were reported in only 26% of the patients and were mainly of focal origin. In our case, focal seizures were the initial neurological symptom, which appeared 1 week earlier than the other symptoms. Seizures have also been reported as an initial symptom of RP in a limited number of cases (31). Although seizures are more common in other connective tissue diseases, such as systemic lupus erythematosus, RP should also be included in the differential diagnosis of new-onset seizures, especially those with suggestive signs such as cauliflower ear and/or red eyes.

Brain MRI findings in patients with RP-associated meningoencephalitis include non-specific white matter changes and preferential involvement of the medial temporal lobes (9). Nine patients have been reported with meningeal enhancement on contrast-enhanced T1-weighted MRI, including those from our center. However, because some cases did not state whether brain MRI was performed with or without contrast, the prevalence of meningeal enhancement was unclear. Calcification and thickening in the ears on CT scans (2, 9) and abnormal DWI and T1-weighted signals in the auricles on MRI scans (9) could also clarify the diagnosis but may easily be neglected.

A CSF analysis is required to rule out infectious diseases and steroid contraindications. In line with a previous study, pleocytosis was common in CSF tests, with a predominance of mononuclear cells (9). A predominance of polymorphonuclear cells was also observed in some cases of marked peripheral inflammation (9). In the cases included in our study, eight patients had a CSF white blood cell count of >500/μL. Among them, four patients showed pleocytosis with a predominance of polymorphonuclear cells. In this scenario, it is more crucial to fully rule out infectious diseases. An increase in CSF protein levels is also very common, and most patients show a mild elevation. The patient from our center demonstrated a normal CSF cell count and mild protein elevation; however, CSF metagenomic next-generation sequencing revealed streptococcus infection. Given that sufficient intravenous ceftriaxone had been administered for more than 1 week before this CSF study, streptococcus or other bacterial meningitis was not considered.

The treatment of RP is largely empirical owing to its rarity, diversity of symptoms, unpredictable recurrence, and potential CNS involvement (9). High-dose or pulse-range steroids are the mainstays of treatment. High-dose steroids should be started immediately when RP is complicated by meningoencephalitis (4). For patients who did not respond to steroids or experienced recurrence during steroid tapering, immunosuppressants such as cyclophosphamide, methotrexate, azathioprine, and mycophenolate mofetil were added. Biologics, such as infliximab (11), rituximab (19), and adalimumab (25), have also been used in a few severe cases with various prognoses. Some studies have suggested that the combined use of methylprednisolone pulse therapy and immunosuppressants is effective in improving prognosis (25). The reported mortality rate of RP-associated meningitis was 12% (3/25) and that of patients with RP-associated encephalitis was 36.4% (4/11) (11). For patients with RP-associated meningoencephalitis in our study, the reported mortality rate was 15% (4/26), with an improvement in 75% (19/26) of the patients. Relapse of RP is common, and therefore, close follow-up is essential for the timely adjustment of the treatment plan. Considering that the patient in our center had COVID-19, steroid pulse therapy was not initiated. Dexamethasone (10 mg/day), which is the recommended steroid regimen for COVID-19, was administered. We were initially very concerned that our immunosuppressive regimen was not strong enough because the patient was gradually declining and very lethargic before the steroid treatment. Fortunately, the patient showed immediate improvement. However, considering the normal CSF cell count and the absence of abnormal parenchymal signals, CNS inflammation in our patient may not have been severe. Therefore, clarifying factors such as peripheral blood inflammation indicators, results of CSF analysis, or neuroimaging findings that may help predict prognosis and guide immunosuppressant selection is an important factor that deserves further research.

This study has several limitations. First, the diagnoses of encephalitis, meningitis, and meningoencephalitis may overlap and be easily confused. Therefore, in our study, we only included cases that were clearly diagnosed with meningoencephalitis. Second, only studies published in English were included, and the sample size was relatively small. Third, a biopsy was not performed on our patient; therefore, we were unable to make a histopathological diagnosis.

## Conclusion

Meningoencephalitis associated with RP is rare but fatal. Our study found that it predominantly affects men, with red or deformed ears being the most common and suggestive feature, although the symptoms can vary. Abnormal parenchymal signals and/or meningeal enhancement can be observed on brain MRI. CSF lymphocytic pleocytosis with mild protein elevation was observed in most of the patients. Recognition of disease characteristics is important because immunosuppressive therapy is effective and should be initiated without delay to achieve a satisfactory prognosis.

## Data availability statement

The datasets presented in this article are not readily available because of ethical and privacy restrictions. Requests to access the datasets should be directed to the corresponding author.

## Author contributions

DZ: Conceptualization, Data curation, Formal analysis, Funding acquisition, Methodology, Visualization, Writing – original draft, Writing – review & editing, Investigation. JS: Data curation, Formal analysis, Investigation, Methodology, Writing – review & editing. XZ: Data curation, Investigation, Methodology, Writing – review & editing. JW: Project administration, Resources, Supervision, Writing – review & editing. YS: Formal analysis, Project administration, Supervision, Writing – review & editing.
